# Malignant lymphoma with diffuse cardiac involvement detected by multiple imaging examinations: a case report

**DOI:** 10.1186/1752-1947-6-193

**Published:** 2012-07-10

**Authors:** Toshiji Ishiwata, Norihiro Harada, Ryo Ko, Munechika Hara, Mitsuaki Sekiya, Makoto Sasaki, Bunsei Nobukawa, Kazuhisa Takahashi

**Affiliations:** 1 Department of Respiratory Medicine, Juntendo University School of Medicine, 2-1-1 Hongo Bunkyo-Ku, Tokyo, 113-8421, Japan; 2 Department of Hematology, Juntendo University School of Medicine, 2-1-1 Hongo Bunkyo-Ku, Tokyo, 113-8421, Japan; 3 Department of Human Pathology, Juntendo University School of Medicine, 2-1-1 Hongo Bunkyo-Ku, Tokyo, 113-8421, Japan

## Abstract

**Introduction:**

In malignant lymphoma, cardiac involvement, which usually forms pathologically focal and firm nodules in the cardiac walls, is considered to be a late manifestation of the disease.

**Case presentation:**

We describe the case of a 71-year-old Asian Japanese woman whose first presentation of lymphoma was congestive heart failure. Multiple imaging examinations and laboratory findings led to a presumed diagnosis of a malignant lymphoma. A tissue diagnosis of the mediastinal mass could not be performed due to our patient’s generally poor condition. Our patient received corticosteroid therapy, but died 42 days after her admission. An autopsy revealed lymphoid cells encircling her ventricular wall and infiltrating her endocardium. A histological examination confirmed the diagnosis of diffuse large B-cell lymphoma.

**Conclusion:**

Imaging examinations such as echocardiography, computed tomography with three-dimensional reconstruction, and gallium-67-citrate scintigraphy could clearly detect the diffuse cardiac involvement antemortem. A combination of these imaging techniques could provide a working diagnosis and allow empirical initiation of treatment in patients with poor general condition.

## Introduction

Cardiac tumors have many clinical presentations and have many differential diagnoses (Table 1). Among these cardiac tumors, metastatic cardiac tumors are relatively more common than primary cardiac tumors. Metastases to the heart were found in 1.23% of 12,485 consecutive autopsies, compared with a 0.056% prevalence of primary cardiac tumors [1-3].

**Table 1:** Differential diagnosis of cardiac tumors

**Benign tumors**	**Malignant tumors**
Myxomas	Sarcomas
Papillary fibroelastomas	Angiosarcoma
Rhabdomyomas	Rhabdomyosarcoma
Fibromas	Fibrosarcoma
Teratomas	Leiomyosarcoma
Purkinje cell tumors	Malignant lymphoma
Lipomas	Pericardial mesothelioma
	Metastatic cardiac tumor
	Bronchogenic carcinoma
	Breast cancer
	Lymphoma
	Melanoma
	Mesothelioma
	Renal cell carcinoma
	Osteosarcoma
	Pericardial mesothelioma

Cardiac involvement of a malignant lymphoma is usually a late manifestation of the disease; therefore, cardiac failure as the initial presentation is extremely rare [4,5]. It is difficult to make a definite diagnosis of these cases, because a surgical biopsy usually cannot be performed due to the risk of cardiac failure. Therefore, most of these patients have died before starting chemotherapy and are only diagnosed postmortem.

A malignant lymphoma with cardiac involvement usually leads to the formation of focal masses in the cardiac wall, and the diffuse involvement pattern is uncommon. Moreover, diffuse cardiac involvement generally cannot be detected by simple imaging examinations such as chest X-ray or usual computed tomography (CT) [6].

We present the case of a patient with a malignant lymphoma with diffuse cardiac involvement whose first presentation was congestive heart failure. A definitive diagnosis was not obtained antemortem because of our patient’s poor general condition. However, a combination of several imaging modalities, including echocardiography, CT with three-dimensional CT reconstruction and nuclear scanning, could detect the diffuse cardiac involvement, and the findings were similar to the autopsy findings.

## Case presentation

A 71-year-old Asian Japanese woman was admitted to our hospital complaining of cough, dyspnea and peripheral edema that had lasted for several weeks. On physical examination, she had a low-grade fever, an irregular heartbeat and hypertension, and an oxygen saturation of 92% with 2L/min via a nasal cannula. Peripheral lymphadenopathy and hepatosplenomegaly were absent.

Our patient’s white blood cell count was 9.4 × 10^9^/L, with 92.0% neutrophils, 3.5% lymphocytes, 4.0% monocytes and 0.5% basophils. She had elevated levels of C-reactive protein (11.6mg/dL; normal range <0.2 mg/dL), soluble interleukin-2 receptor (2,220U/mL, normal range: 145 to 519U/mL) and N-terminal pro-B-type natriuretic peptide (2,795.0pg/mL; normal range <125.0pg/mL).

**Figure 1:**
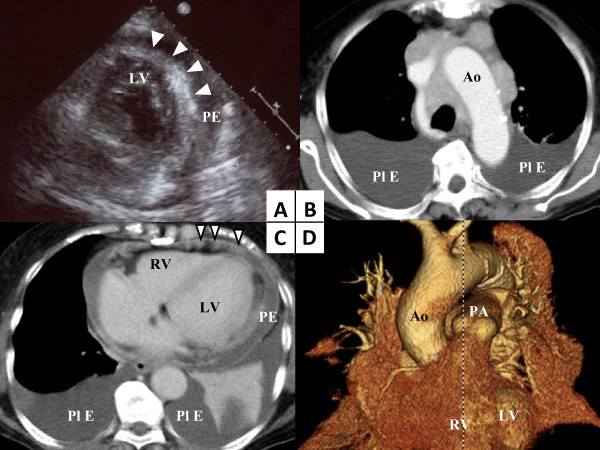
**Imaging studies. (A)** Echocardiography revealed a high echoic region outside the ventricular wall (arrow head). **(B, C)** The computed tomography scan on admission revealed a mediastinal mass, pericardial nodules (arrow head), and pericardial and bilateral pleural effusions. **(D)** Three-dimensional computed tomography reconstruction imaging revealed the rough surface of the epicardium. Ao: aorta; LV: left ventricle; PA: pulmonary artery; PE: pericardial effusion; Pl E: pleural effusion; RV: right ventricle.

Virological tests related to myocarditis were negative and her angiotensin-converting enzyme level, associated with sarcoidosis, was normal. Electrocardiography showed atrial fibrillation but no evidence of any ischemic changes. Furthermore, echocardiography showed normal systolic function of her left ventricle (ejection fraction of 65%) though the diastolic function was moderately impaired (pseudonormal) due to an impairment of myocardial relaxation [7]: the ratio of the peak early diastolic filling velocity to the peak atrial filling velocity (E/A ratio) was increased to 1.6 in spite of the advanced age of our patient. The deceleration time did not decrease (174msec). The peak early diastolic velocity (e’) did decrease to 8.2cm/s, while the ratio of the peak early diastolic filling velocity to the peak early diastolic velocity (E/e’ ratio) increased to 11.0. In addition, echocardiography revealed a diffuse and high echoic mass outside of the ventricular wall with pericardial effusion, which was originally thought to be a fibrin clot (Figure 1A, arrow head).

Chest X-rays showed bilateral pleural effusion with mediastinal widening. A CT scan of her body trunk was performed to better characterize the lesion anatomically, and this revealed a mediastinal mass and pericardial nodules, as well as pericardial and pleural effusion (Figure 1B, C). Additionally, three-dimensional CT reconstruction (Figure 1D) showed the presence of a rough surface over the whole epicardium. These CT imaging findings showed no mass-like lesions on bilateral lung fields or within the thoracic wall.

**Figure 2:**
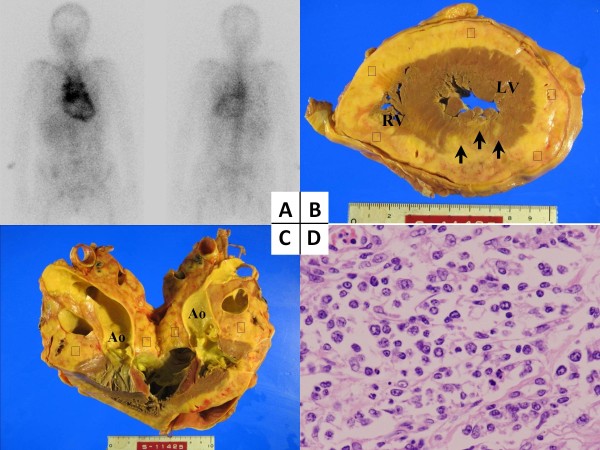
**Gallium-67-citrate scintigraphy and postmortem specimen examination. (A)** Anterior (left) and posterior (right) ^67^Ga scans showed increased uptake by only the thoracic lymph nodes and the heart. (**B, C)** The cut surface of the heart at autopsy revealed lymphomatous involvements (*) of the epicardium (B) and the mediastinum (C). Of note, some of the involvements had infiltrated into the endocardium (arrow). **(D)** A histological section of the mediastinal lymph node demonstrated dense proliferation of large centroblastic cells with abundant pale cytoplasm. (Hematoxylin and eosin stain ×100.) Ao: aorta; LV: left ventricle; RV: right ventricle.

We next performed gallium-67-citrate (^67^Ga) scintigraphy to test for the existence of lymph nodes in order to make a tissue diagnosis. The ^67^Ga uptake was observed in the mediastinal lymph nodes, but not in other lymph nodes. Interestingly, ^67^Ga uptake was diffusely seen in the heart (Figure 2A).

Pericardial and bilateral pleural effusions were drawn and found to be bloody, with an exudate pattern and no malignant findings on cytology. A bacterial culture revealed no microorganisms. Additionally, a flow cytometric analysis of both types of effusions showed predominant lymphocytes (B-cell to T-cell ratio of 1:4); however, there was no monoclonal pattern.

A surgical biopsy of the mediastinal mass was considered to achieve a pathological diagnosis. However, because our patient’s respiratory condition was extremely poor, neither the surgical biopsy, including thoracoscopy, nor the transarterial catheter biopsy could be performed. The most probable diagnosis was a malignant lymphoma because of the presence of a mediastinal mass and an elevated soluble interlukin-2 receptor level, which is elevated in patients with non-Hodgkin lymphoma [8]. Furthermore, differential diagnoses, such as myocarditis, tuberculosis and other malignant tumors, bronchogenic carcinoma, mesothelioma and melanoma were extremely unlikely based on the results of the laboratory and imaging tests. Our patient was treated with only 25mg of prednisolone, which is one of the antitumor medicines for a malignant lymphoma, due to her poor performance status and undiagnosed condition. However, the corticosteroid therapy failed to reduce the tumor size at least until day 16, when our patient’s condition gradually deteriorated and she subsequently died of cardiac failure. An autopsy was performed after permission was obtained from her family. The macroscopic evaluation during the autopsy revealed that the mediastinal mass consisted of a cluster of enlarged lymph nodes. A number of disseminated masses were found on the pericardial membrane. These involvements had encircled the ventricle walls and penetrated through the cardiac layers to her endocardium (Figure 2B, C).

On microscopy, the mediastinal lymph nodes demonstrated a complete effacement of the normal architecture because of the dense proliferation of large blastoid cells. These cells resembled normal centroblasts, and showed large and non-cleaved cells with vesicular chromatin (Figure 2D). The immunohistochemical staining showed that the tumor cells were positive for cluster of differentiation (CD)20, CD79a, paired box 5 and B-cell lymphoma 2, and negative for CD3, CD4, CD5, CD8, CD45RO, CD56, multiple myeloma oncogene 1 and cyclin D1. Accordingly, the final diagnosis was established as mediastinal diffuse large B-cell lymphoma. The presumed cause of death was the impairment of cardiac function due to such lymphomatous involvement of the heart.

## Discussion

Cardiac involvement is observed in around 20% of patients with advanced stage malignant lymphoma, according to a series of autopsies [9]. However, cases with cardiac involvement that show cardiac failure at initial presentation are extremely rare, requiring a high level of suspicion for diagnosis, and result in a poor prognosis for the patient [4]. Cardiac involvement is generally reported to include the formation of focal and firm nodules in the cardiac walls and/or the pericardium. In our case, the cardiac involvements did not show any nodular lesions, but the cardiac wall was diffusely encircled. Diffuse cardiac involvement of a malignant lymphoma has been reported in only a few cases [6,10,11]. Most of them showed some signs of cardiac failure as the initial symptom, and could not be diagnosed antemortem, as in our case [10,11]. Based on these reports, diffuse cardiac involvement is typically associated with uniform infiltration into the myocardium. However, to the best of our knowledge, diffuse cardiac involvements encircling the ventricle, as in our patient, has not yet been reported. Imaging examinations such as echocardiography, CT (including three-dimensional CT reconstruction) and nuclear scanning were employed in these cases to detect the cardiac involvements.

Echocardiography has been reported to be a useful examination for evaluating the cardiac involvement of malignant lymphomas showing a mass lesion, pericardial effusion and thickened ventricular wall [4]. Echocardiography detected the cardiac involvement in our case as well. A high echoic mass outside of the ventricle encircled our patient’s heart. Additionally, the diastolic dysfunction of her left ventricle was revealed, caused by impaired myocardial relaxation. These echocardiographic findings have not yet been reported in other cases. Therefore, echocardiography may also be useful for detecting cardiac involvement in terms of imaging the inner structure of the cardiac wall and also for evaluating cardiac function.

In contrast, CT scanning did not provide clear images of the inner structure of the cardiac wall. However, CT scans could detect mediastinal lymphadenopathy and, in particular, three-dimensional CT reconstruction could clearly reveal the rough surface of the epicardium. In addition, three-dimensional CT reconstruction can display stereoscopic images that are useful for understanding the spatial relationship between the tumor and the anatomical structures, as was demonstrated in our case. Therefore, three-dimensional CT reconstruction is a useful imaging method for determining the cardiac involvement of a malignant lymphoma. To the best of our knowledge, the utility of three-dimensional CT reconstruction for cardiac involvement of a malignant lymphoma has not yet been reported. ^67^Ga scintigraphy, a nuclear scanning examination, could detect diffuse infiltration of the heart. However, the diagnostic sensitivity of nuclear scanning was lower than that of CT because of the low-resolution nature of the images. Therefore, it is important to combine nuclear scanning with other imaging methods when assessing the lymphomatous involvement of the heart. In recent years, ^18^F-Fluorodeoxyglucose positron-emission tomography (^18^FDG-PET), which is another form of nuclear scanning, appears to be more sensitive for detecting nodal and extranodal involvements than ^67^Ga scintigraphy [12]. Furthermore, CT and ^18^FDG-PET have been recommended for the pre- and post-treatment evaluation of nodal diffuse large B-cell lymphoma because of their high sensitivity [13]. However, ^18^FDG-PET scanning could not be conducted in this case because it could not be performed in our hospital and our patient’s general condition was too serious for transfer to another hospital. ^67^Ga scanning, which was formerly the standard technique, could detect the lymphomatous involvements of the heart [12]. Therefore, nuclear scanning should be performed in cases of suspected lymphoma.

The imaging modalities we employed provided, in conjunction, clear evidence of the cardiac involvement and raised a reasonable suspicion of a malignant lymphoma. In light of our patient’s poor condition that precluded a pathologic diagnosis, we were compelled to act upon this suspected diagnosis and initiated corticosteroid therapy. While in the case of our patient, the intervention was not able to prolong survival, a younger patient in better general health might have benefited from such a diagnosis.

## Conclusion

The patient presented here had a rare case of malignant lymphoma with diffuse cardiac involvement that initially presented as cardiac failure. Diffuse cardiac involvement of malignant lymphoma may result in cardiac dysfunction and a poor prognosis. Some imaging techniques can be noninvasively applied in these serious cases and correctly image the cardiac involvement. Echocardiography can detect cardiac involvement, including the inner structure of the cardiac wall and the impairment of cardiac function caused by these structures; CT with three-dimensional reconstruction can survey the lymphomatous involvement, including the epicardial surface; and nuclear scanning can provide information suggesting whether or not the mass is malignant. A combination of these imaging techniques can strongly suggest lymphomatous involvement of the heart, providing a working diagnosis and allowing empirical initiation of treatment.

## Consent

Written informed consent was obtained from the patient’s next-of-kin for publication of this manuscript and any accompanying images. A copy of the written consent is available for review by the Editor-in-Chief of this journal.

## Abbreviations

CD, cluster of differentiation; CT, Computed tomography; 67 Ga, gallium-67-citrate; 18FDG-PET, 18F-Fluorodeoxyglucose positron-emission tomography.

## Competing interests

The authors declare that they have no competing interests.

## Authors’ contributions

TI, NH and MSa reviewed the clinical data and were major contributors in writing the manuscript. RK, MH, MSe and KT were involved with patient management. BN analyzed histological data and performed the immunohistochemical analysis. All authors read and approved the final manuscript.
